# Chlorotyrosine protein adducts are reliable biomarkers of neutrophil-induced cytotoxicity *in vivo*

**DOI:** 10.1186/1476-5926-2-S1-S48

**Published:** 2004-01-14

**Authors:** Jaspreet S Gujral, Jack A Hinson, Hartmut Jaeschke

**Affiliations:** 1 Department of Pharmacology and Toxicology, University of Arkansas for Medical Sciences, Little Rock, Arkansas 72205, USA; 2 Liver Research Institute, University of Arizona, Tucson, Arizona 85724, USA

## Abstract

**Introduction:**

A limitation for investigating the pathophysiological role of neutrophils *in vivo *is the lack of a reliable biomarker for neutrophil cytotoxicity in the liver. Therefore, we investigated if immunohistochemical detection of chlorotyrosine protein adducts can be used as a specific footprint for generation of neutrophil-derived hypochlorous acid *in vivo*.

**Methods:**

C3Heb/FeJ mice were treated with 100 micrograms/kg endotoxin (ET) alone or in combination with 700 mg/kg galactosamine (Gal/ET). Some animals received additionally two doses of 10 mg/kg of the pancaspase inhibitor Z-VAD-fmk. An antibody against chlorotyrosine was used for the immunohistochemical analysis.

**Results:**

At 6 h after Gal/ET, hepatocellular apoptosis was evident without increase in plasma ALT activities. Neutrophils accumulated in sinusoids but there was no evidence for chlorotyrosine staining. At 7 h after Gal/ET, about 54% of the sequestered neutrophils had extravasated, there was extensive necrosis and increased plasma ALT activities. Extensive immunostaining for chlorotyrosine, mainly colocalized with neutrophils, could be observed. Treatment with Z-VAD-fmk eliminated apoptosis, necrosis and the increase in plasma ALT values. Neutrophil extravasation was prevented but the overall number of neutrophils in the liver was unchanged. Chlorotyrosine staining was absent in these samples. After ET alone (7 h), sinusoidal neutrophil accumulation was similar to Gal/ET treatment but there was no apoptosis, neutrophil extravasation, ALT release or chlorotyrosine staining.

**Conclusions:**

Chlorotyrosine staining in liver samples correlated well with evidence of neutrophil-induced liver injury in the endotoxemia model. These results indicate that assessment of chlorotyrosine protein adduct formation by immunohistochemistry could be a useful marker of neutrophil-induced liver cell injury *in vivo*.

## Introduction

Neutrophils are involved in the pathophysiology of hepatic ischemia-reperfusion injury, endotoxin- and sepsis-induced liver failure, alcoholic hepatitis, and certain drug toxicities [1]. Prerequisite for neutrophil cytotoxicity is the accumulation in sinusoids, extravasation and the adherence to the parenchymal cells [2]. Neutrophils cause cell injury by generation of reactive oxygen species and protease release [3-6]. A limitation for investigating the pathophysiological role of neutrophils *in vivo *is the lack of a reliable biomarker for neutrophil cytotoxicity in the liver. Neutrophils generate superoxide with NADPH oxidase. They also release myeloperoxidase at the same time and, therefore, form hypochlorous acid as a major oxidant [7]. Hypochlorous acid is a potent chlorinating agent which can cause the formation of chlorotyrosine protein adducts [7]. Antibodies can be generated to detect chlorotyrosine in the tissue [8,9]. However, this approach has never been validated in models of neutrophil-induced liver injury *in vivo*. Therefore, we investigated if immunohistochemical detection of chlorotyrosine protein adducts can be used as a specific footprint for generation of neutrophil-derived hypochlorous acid *in vivo*. To test this hypothesis, we used the well-characterized model of galactosamine/endotoxin (Gal/ET)-induced liver injury, where neutrophil cytotoxicity aggravates the initial apoptotic injury [10], by a reactive oxygen-dependent mechanism [3].

## Results and Discussion

Neutrophil accumulation in the hepatic sinusoids and extravasation into the parenchymal tissue has been shown to occur after Gal/ET treatment in mice [2,4]. In our study, immunohistochemical analysis of liver sections showed that neutrophils accumulated in the livers after Gal/ET treatment (6 h: 202 – 21 per 10 high power fields, HPF; 7 h: 343 – 28 per 10 HPF). At 6 h most of these neutrophils (about 90%) remained in the sinusoids. The plasma alanine transaminase (ALT) activities at this time did not increase over controls. However, by 7 h post-treatment, over 50% of the neutrophils present in the livers had extravasated into the parenchyma (Table 1). The plasma ALT activities increased 20-fold over untreated controls (Table 1). Histological evaluation of necrosis in hematoxylin and eosin (H&E)-stained sections revealed 45% necrotic hepatocytes at 7 h compared to 15% at 6 h (Table 1).

**Table 1:** Neutrophil extravasation, liver injury and chlorotyrosine adduct formation during murine endotoxemia.

	Number of Extravasated Neutrophils (per 10 HPF)	Plasma ALT Activities (U/L)	Necrosis (%)	Chlorotyrosine Staining (Intensity)
Controls	0 – 0	64 – 16	0 – 0	0
Gal/ET 6 h	23 – 3	41 – 9	15 – 2*	0
Gal/ET 6 h + ZVAD	6 – 2	18 – 13	2 – 1^#^	0
Gal/ET 7 h	186 – 30*^,$^	1252 – 369*^,$^	45 – 2*^,$^	++++
Gal/ET 7 h + ZVAD	19 – 5^#^	10 – 3^#^	3 – 1^#^	+

Animals were sacrificed 6 or 7 h after Gal/ET treatment. Some animals received 2 doses of the pancaspase inhibitor ZVAD (10 mg/kg) at 3 and 4.5 h. Alanine aminotransferase (ALT) activities were measured in plasma. Liver sections were immunostained with the anti-Gr1 and anti-chlorotyrosine antibodies for evaluation of neutrophil extravasation and chlorotyrosine adduct formation, respectively. Necrosis was estimated in sections stained with H&E. Data represent means – SE of n = 4 animals per group. *P &lt; 0.05 (compared to controls) ^$^*P *&lt; 0.05 (compared to Gal/ET6 h) ^#^P &lt; 0.05 (compared to Gal/ET alone).

**Figure 1:**
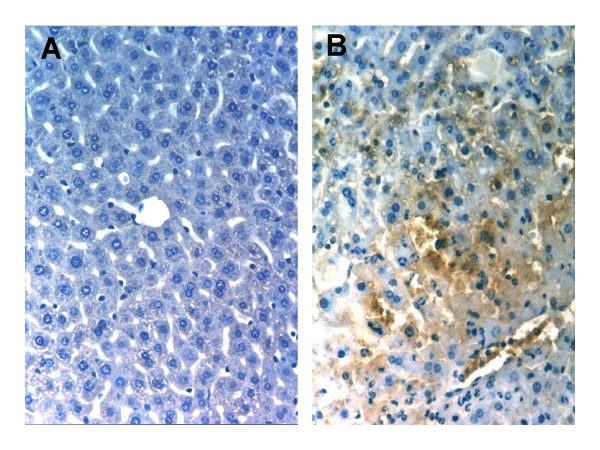
**Chlorotyrosine-protein adduct formation in the liver. **Liver sections were immunostained with an anti-chlorotyrosine antibody to assess the formation of chlorotyrosine-protein adducts after galactosamine/endotoxin treatment. No staining was observed in livers from untreated controls (A). After Gal/ET treatment for 7 h, the liver sections showed extensive positive staining for adducts inside hepatocytes as well as along sinusoids (B).

Liver sections were immunostained with a rabbit anti-chlorotyrosine antibody to assess the formation of chlorotyrosine-protein adducts in the liver. No positive staining was observed in control livers (Figure 1) or after Gal/ET 6 h (Table 1). After Gal/ET treatment for 7 h, the liver sections showed extensive positive staining for the adducts inside the hepatocytes as well as along the sinusoids (Figure 1). Moreover, sequential staining with the anti-chlorotyrosine and -Gr1 antibodies of the same liver sections showed co-localization of the chlorotyrosine adducts with the extravasated neutrophils. On the other hand, liver sections of mice treated with endotoxin alone for 7 h (ET 7 h) showed only slight positive staining in the sinusoids.

Gal/ET treatment causes caspase activation and hepatocellular apoptosis [10]. To confirm this, hepatic caspase-3 activities were measured using a synthetic fluorogenic caspase-3 substrate, Ac-DEVD-MCA. Compared to controls, caspase-3 activities increased by 365-fold and 600-fold at 6 h and 7 h, respectively. Hepatocellular apoptosis was quantified, using morphological criteria, in liver sections stained with the terminal deoxynucleotidyl transferase-mediated dUTP nick end labeling (TUNEL) assay, and expressed as a percentage of the total cells evaluated [11,12]. Apoptotic cells were rare in control livers (&lt;0.1%). After Gal/ET treatment, hepatocellular apoptosis increased to 13% at 6 h and 19% at 7 h.

If parenchymal cell apoptosis is prevented by various caspase inhibitors [10] or by injection of endotoxin without galactosamine [2], transmigration of neutrophils does not occur and any injury is eliminated. Therefore, in order to investigate the effect of a pancaspase inhibitor on neutrophil-induced cytotoxicity and chlorotyrosine adduct formation, animals were treated with Z-VAD-fmk in addition to Gal/ET. Z-VAD reduced the hepatic caspase-3 activities to baseline and the hepatocellular apoptosis at 6 and 7 h by 90%. It also reduced the number of extravasated neutrophils in the tissue and liver cell injury (plasma ALT and necrosis) at 7 h, by 90% (Table 1). This was accompanied by reduced immunostaining for chlorotyrosine adducts inside the hepatocytes. Only mild positive staining was observed in the sinusoids around the neutrophils. However, the pancaspase inhibitor did not affect the total number of neutrophils accumulated in the livers at both time points, i.e., 6 h and 7 h after Gal/ET.

These data show that the formation of chlorotyrosine protein adducts closely follows the extravasation of neutrophils (Gal/ET 7 h). Protein adduct formation also correlated well with the dramatic increase in the plasma ALT levels and liver cell necrosis as indicators for cytotoxicity of neutrophils. These findings are consistent with the hypothesis that the activated neutrophils generated hypochlorous acid. In contrast, no adducts were formed during the early phase of the apoptotic injury, i.e., when 90% of the neutrophils in the liver remained inactive in the sinusoids (Gal/ET 6 h). Likewise, no protein adducts were formed when the neutrophils were primed but not fully activated (ET 7 h), or when the chemotactic signal for neutrophil extravasation was removed by elimination of the apoptotic cell injury (Gal/ET 7 h + ZVAD). Neutrophils can mediate tissue injury either through reactive oxygen species or the release of proteases [3-6]. In this regard, our study further supports the role of reactive oxygen species, and hypochlorous acid in particular, in neutrophil-induced cytotoxicity *in vivo*.

## Conclusions

Chlorotyrosine-protein adducts are formed in the liver during murine endotoxemia, a well-established model of neutrophil-induced liver injury *in vivo*. These adducts can be easily and reliably detected through immunohistochemistry and, thus, can serve as a valuable biomarker for neutrophil-induced cytotoxicity.

## Methods

### Animals

Male C3Heb/FeJ mice (Jackson Laboratories, Bar Harbor, ME) were treated i.p. with 100 micrograms/kg *Salmonella abortus equi *endotoxin (ET) alone or in combination with 700 mg/kg galactosamine (Gal/ET; Sigma Chemical Co., St. Louis, MO). Animals were sacrificed 6 or 7 h after treatment. Some animals received additionally two 10 mg/kg doses of the pancaspase inhibitor Z-VAD-fmk (Enzyme Systems Products, Dublin, CA) at 3 and 4.5 h after Gal/ET. An antibody against chlorotyrosine was generated by standard procedures and used for the immunohistochemical analysis [13]. The following parameters were measured as previously described: caspase-3 activity [10], DNA strand breaks with terminal deoxynucleotidyl transferase-mediated dUTP nick end labeling (TUNEL staining) [11,12], neutrophil localization (anti-Gr1 immunostaining), plasma ALT activities [10], imunohistochemistry for chlorotyrosine adducts, and histological assessment of necrosis in liver sections stained with H&E [11,12].
